# Prevalence of osteo-renal impairment in the Romanian HIV cohort

**DOI:** 10.1186/s12879-016-1397-2

**Published:** 2016-03-08

**Authors:** Anca Streinu-Cercel, Oana Săndulescu, Gabriela Ceapraga, Daniela Manolache, Monica Andreea Stoica, Liliana Lucia Preoțescu, Adrian Streinu-Cercel

**Affiliations:** Carol Davila University of Medicine and Pharmacy, Bucharest, Romania; National Institute for Infectious Diseases “Prof. Dr. Matei Balș”, Bucharest, Romania

**Keywords:** Bone mineral density, DXA, eGFR, Chronic kidney disease, HIV

## Abstract

**Background:**

The Romanian HIV cohort has certain particularities that render it unique in Europe. We have performed a study to evaluate the prevalence of bone and kidney impairment in this particular group of HIV-infected patients.

**Methods:**

We performed dual-energy X-ray absorptiometry (DXA) evaluation of the lumbar vertebrae and the femur, as well as laboratory tests including standard serum panels, bone-related markers and urinalysis in patients from the Romanian HIV cohort.

**Results:**

The study included 72 patients, of which 46 (58.3 %) were males. The median (IQR) age was 38 (18) years and the median (IQR) time from HIV infection diagnosis was 9 (13) years. Most patients (55.6 %) were non-smokers, but a relatively high proportion (37.5 %) was currently smoking. Only a small percentage of patients (20.8 %) did not present any comorbidities, while 40.3 % had one comorbidity, the most frequent being dyslipidemia (present in 25 patients, 38.5 %). Only 6 patients had a medical history suggestive for renal disease and 3 for bone-related abnormalities.

The median (IQR) glomerular filtration rate was 97.5 (33.0) mL/min/1.73sqm. We diagnosed 21 patients (29.6 %) with stage 2 chronic kidney disease and one patient (1.4 %) with stage 3 chronic kidney disease. Proteinuria was present in 9 (12.7 %) patients. The estimated glomerular filtration rate was significantly lower in patients with cardiac comorbidities (*p* = 0.013).

Vitamin D was significantly lower in smokers compared with non-smokers, with a mean value of 15 vs. 21 ng/mL and a moderate effect size (Cohen’s d = −0.5) (*p* = 0.046). Lumbar osteopenia and osteoporosis were diagnosed in 33.3 and 13.7 % of patients, while femoral osteopenia and osteoporosis were diagnosed in 37.3 and 7.8 %, respectively. Lower nadir CD4 cell counts were found in patients with bone-related comorbidities (*p* = 0.000).

**Conclusions:**

We identified a relatively high prevalence of chronic kidney disease in the Romanian HIV cohort, and a fairly low prevalence of osteopenia and osteoporosis, compared with other European countries. In this category of patients smoking should be avoided altogether, as it may be an indirect risk factor for kidney disease (associating cardiac comorbidities) and it may impair bone metabolism by altering serum levels of hydroxy-vitamin D.

## Background

The Romanian HIV cohort has certain particularities that render it unique in Europe. The circulating HIV strains belong mainly to clade F and infection was acquired at an early age, in infancy and childhood, in the late ‘80s [1]. Therefore, patients from the Romanian cohort have seen antiretroviral (ARV) drugs come and go, and they have tried all available therapeutic options, even before the HAART era, when combination therapy had not yet been deemed as standard of care [2].

Given this unique background, and the patients’ history of over 20 years of HIV infection [3], we performed a study to check for renal impairment and bone demineralization in patients monitored for HIV infection in one Romanian HIV reference center in Bucharest [4], in order to design individualized therapeutic approaches and updated guidelines for screening and management in this particular patient population [5, 6].

## Methods

We performed a cross-sectional study in patients from the Romanian HIV cohort, to evaluate renal function through urinalysis, serum creatinine, serum urea, creatinine clearance and estimated glomerular filtration rate (eGFR), calculated using the Modification of Diet in Renal Disease (MDRD) formula [7]. Renal stiffness was assessed by one trained operator through shear-waves elastography on Aixplorer (SuperSonic Imagine, Aix-en-Provence, France). The bone was evaluated through dual-energy X-ray absorptiometry (DXA) scan of the lumbar vertebrae and the left femur. Bone-related markers such as serum hydroxy-vitamin D and parathyroid hormone levels were also quantified. Serum calcium levels but not serum phosphorus levels were assessed.

The Romanian HIV cohort currently includes 13 536 patients (data current through 30 September 2015) [8], from a total of 20 968 patients diagnosed and monitored between 1985 and 2015. Of these, approximately 2200 are under active surveillance in the National Institute for Infectious Diseases “Prof. Dr. Matei Balș”, Bucharest, and 72 patients presented for evaluation and accepted to participate in this study between January 2015 and August 2015.

Prior to inclusion in the study, all patients signed a written informed consent form. The study protocol was approved by the local Ethics Committee.

Statistical analysis was performed with SPSS Statistics for Windows (version 22.0, IBM Corp, Armonk, NY, USA). The descriptive analysis presents the mean and standard deviation (SD) for normally-distributed data or the median and interquartile range (IQR) values for non-normally-distributed data. The statistical analysis reports the independent-samples *t* test with effect size calculation for normally-distributed variables, and the Mann-Whitney U non-parametric test for independent samples, for non-normally-distributed variables. *P* values were considered statistically significant when < 0.05.

## Results

The study included 72 patients, of which 46 (58.3 %) were males. The median (IQR) age was 38 (18) years and the median (IQR) time from HIV infection diagnosis was 9 (13) years. Patient characteristics are presented in Table 1. The mean ± SD weight and height were 71 ± 15.3 kg and 171 ± 9.8 cm, respectively, with a mean ± SD body mass index of 23.7 ± 3.7 kg/sqm. At the time of evaluation, most of the patients had received one prior ARV regimen (31.9 %) or over three prior regimens (30.6 %), while only 3 subjects were at their first treatment regimen (4.2 %). Most patients (54.2 %) had detectable and quantifiable HIV viral loads, while only 38.9 % had undetectable HIV viral loads. The median (IQR) current CD4 cell count was 508 (380) cells/cmm, with a nadir count of 387 (290) cells/cmm, in the range of 19–1190 cells/cmm.

**Table 1:** Patient characteristics

Characteristic	Sub-category	*n* (%)	Median (IQR)	Percentiles 25, 75
Male gender		46 (58.3)		
Age, years			38 (18)	26, 44
Time since HIV diagnosis, years			9 (13)	4, 17
Time since HIV diagnosis, years	1	10 (13.9)		
	2–5	15 (20.8)		
	6–10	13 (18.1)		
	11–15	12 (16.7)		
	16–20	14 (19.4)		
	21–27	8 (11.1)		
Number of prior ARV regimens (*n* = 67)	0	3 (4.5)		
	1	23 (34.3)		
	2	18 (26.9)		
	3	1 (1.5)		
	>3	22 (32.8)		
Current CD4 cell count, cells/cmm			508 (380)	289, 669
Nadir CD4 cell count, cells/cmm			387 (290)	229, 519
Current viral load, copies/mL	Undetectable	28 (38.9)		
	Below LLQ^a^	5 (6.9)		
	Detectable (range: 27 to 170,000,000)	39 (54.2)	5617 (95224)	37.5, 95261
Smoking status	Ex-smoker^b^	2 (2.8)		
	Current-smoker	27 (37.5)		
	Non-smoker	40 (55.6)		
	Refused to declare	3 (4.2)		
Number of comorbidities (*n* = 65)	0	15 (20.8)		
	1	29 (40.3)		
	2	13 (18.1)		
	3	2 (2.8)		
	4	5 (6.9)		
Type of comorbidities (*n* = 65)	Cardiac	5 (7.7)		
	Metabolic	28 (43.1)		
	Renal	6 (9.2)		
	Bone-related	3 (4.6)		
	Hemato-oncologic	5 (7.7)		

*ARV* antiretroviral, *IQR* interquartile range, *LLQ* lower limit of quantitation

^a^HIV-RNA TaqMan – limit of detection of 20 copies/mL

^b^Ex-smokers were defined as having completely quit smoking for over 6 months

Most patients (55.6 %) were non-smokers, but a relatively high proportion (37.5 %) was currently smoking. Only a small percentage of patients (20.8 %) did not present any comorbidities, while 40.3 % had one comorbidity, the most frequent being dyslipidemia (present in 25 patients, 38.5 %). Thirteen patients (18.1 %) had 2 comorbidities, 2 patients (2.8 %) displayed 3 comorbidities and 5 patients (6.9 %) had 4 comorbidities. Importantly, only 6 patients had a medical history suggestive for renal disease: renal cyst (*n* = 3), renal angiomyolipoma (*n* = 1), renal lithiasis (*n* = 2), and hydronephrosis (*n* = 1). Interestingly, of these 6 patients 4 had normal eGFR (and negative proteinuria), while the other two patients presented stage 1 and 3 chronic kidney disease, respectively. Three patients had positive medical history for bone-related abnormalities (herniated lumbar disc, bilateral coxarthrosis, and rheumatoid arthritis, respectively). Of these, two had insufficient serum levels of hydroxy-vitamin D, abnormal (high/low) parathyroid hormone levels but normal bone mineralization, while the third patient had normal serum levels of hydroxy-vitamin D and parathyroid hormone but was diagnosed with femoral osteopenia.

One patient refused to undergo urinalysis or serum screening for kidney function. For the rest of the patients, the median (IQR) eGFR estimated by MDRD was 108.4 (34.7) mL/min/1.73sqm (Table 2). Based on our screening procedures, 21 patients (29.6 %) were diagnosed with stage 2 chronic kidney disease (with a mild decrease in eGFR, in the range of 60–89.9 mL/min/1.73 sqm) and one patient (1.4 %) with stage 3 chronic kidney disease (eGFR of 59.5 mL/min/1.73 sqm). Proteinuria was present in only 9 (12.7 %) of patients.

**Table 2:** Kidney function tests results for patients screened in this study (*n* = 71)

Characteristic	Sub-category	*n* (%)	Median (IQR)	Percentiles 25, 75	Mean ± SD
Serum urea, mg/dL			N/A	N/A	30.2 ± 8.5
Serum creatinine, mg/dL			0.8 (0.3)	0.7, 1.0	N/A
eGFR, mL/min/1.73 sqm			108.4 (34.7)	89.5, 124.2	N/A
eGFR	Stage 1 > 90	49 (69.0)			
	Stage 2 60–89.9	21 (29.6)			
	Stage 3 30–59.9	1 (1.4)			
	Stage 4 15–29.9	0 (0)			
	Stage 5 < 15	0 (0)			
Positive proteinuria, *n* (%)		9 (12.7)			
Renal elastography, kPa			17.1 (11)	12.4, 23.4	N/A

*eGFR* estimated glomerular filtration rate, *IQR* interquartile range, *N/A* not applicable, *SD* standard deviation

eGFR was calculated by MDRD [7] and is expressed in mL/min/1.73 sqm. Renal stiffness was assessed for 19 patients by one trained operator through shear-waves elastography on Aixplorer (SuperSonic Imagine, Aix-en-Provence, France)

The eGFR did not differ significantly with gender (*p* = 0.523), smoking status (*p* = 0.109), number of comorbidities (*p* = 0.387), presence of bone-related comorbidities (*p* = 0.871), kidney (*p* = 0.290), metabolic (*p* = 0.160) or hemato-oncologic comorbidities (*p* = 0.541). However, eGFR was significantly lower in patients with cardiac comorbidities (*p* = 0.013, U = 52).

Bone-related markers were measured for 63 patients who consented to the blood tests. Hypoparathyroidism was diagnosed in 7.9 % of patients, and hyperparathyroidism in 33.3 %. Most patients (65.1 %) had insufficient serum levels of hydroxy-vitamin D, and 25.4 % had hydroxy-vitamin D deficiency (Table 3). Notably, screening was performed during spring and summer. Vitamin D was significantly lower in smokers compared with non-smokers, with a mean value of 15 vs. 21 ng/mL and a moderate effect size (Cohen’s d = −0.5) (*p* = 0.046).

**Table 3:** Bone-related tests results for patients screened in this study

Characteristic	Sub-category	*n* (%)	Median (IQR)	Percentiles 25, 75	Mean ± SD
DXA scan result (*n* = 51)	Normal bone mineral density	23 (45.1)			
	Lumbar osteopenia	17 (33.3)			
	Lumbar osteoporosis	7 (13.7)			
	Femoral osteopenia	19 (37.3)			
	Femoral osteoporosis	4 (7.8)			
Lumbar L1-L4 DXA T-score			N/A	N/A	–0.8 ± 1.0
Left femur DXA T-score			N/A	N/A	–0.4 ± 0.8
Serum parathyroid hormone, pg/mL			48.2 (43.6)	27.7, 71.3	N/A
Interpretation of serum parathyroid hormone (*n* = 63)	Decreased (<15 pg/mL)	5 (7.9)			
	Normal (15–65 pg/mL)	37 (58.7)			
	Increased (>65 pg/mL)	21 (33.3)			
Serum hydroxy-vitamin D, ng/mL			N/A	N/A	18.8 ± 10.8
Interpretation of serum hydroxy-vitamin D levels (*n* = 63)	Deficient (<10 ng/mL)	16 (25.4)			
	Insufficient (10–29.9 ng/mL)	41 (65.1)			
	Optimal (30–99.9 ng/mL)	6 (9.5)			
	Toxic (>100 ng/mL)	0 (0)			

*IQR* interquartile range, *N/A* not applicable, *SD* standard deviation

DXA scans were performed in 51 patients; the rest of the patients did not consent to the scan. Almost half (45.1 %) of the results were suggestive for normal bone mineral density, but a high proportion of patients already displayed osteopenia (33.3 % with lumbar and 37.3 % with femoral localization). Furthermore, lumbar osteoporosis was diagnosed in 13.7 % of patients (with ages of 26, 30, 33, 38, 40, 47 and 65 years) and femoral osteoporosis in 7.8 % of patients (with ages of 26, 40, 44, 50 years).

Lumbar or femoral T-scores did not differ significantly with gender (*p* = 0.850 and *p* = 0.961, respectively), number of comorbidities (*p* = 0.168 and *p* = 0.126, respectively), presence of bone-related comorbidities (*p* = 0.933 and *p* = 0.826, respectively), kidney (*p* = 0.489 and *p* = 0.750, respectively), metabolic (*p* = 0.291 and *p* = 0.394, respectively), cardiac (*p* = 0.828 and *p* = 0.343, respectively) or hemato-oncologic comorbidities (*p* = 0.306 and *p* = 0.618, respectively).

We identified a statistical association between bone-related comorbidities and nadir CD4 cell count, patients with this type of comorbidities having lower mean nadir CD4 cell counts (150 vs. 406 cells/cmm), with a large effect size (Cohen’s d = −1.5). No other statistical associations were found between other types of comorbidities and current or nadir CD4 cell count, HIV viral load, renal stiffness, hydroxy-vitamin D or parathyroid hormone (Table 4).

**Table 4:** Statistical associations between type of comorbidities and laboratory parameters

Type of comorbidities	Current CD4 cell count	Nadir CD4 cell count	HIV viral load	Renal stiffness	Hydroxy-vitamin D	Parathyroid hormone	Smoking status
Cardiac	0.472	0.685	0.321	0.443	0.910	0.053	0.052
Metabolic	0.136	0.774	0.326	0.553	0.579	0.091	0.090
Renal	0.275	0.362	0.321	N/A^a^	0.365	0.532	1.000
Bone-related	0.969	**0.000**	0.326	0.443	0.500	0.613	0.324
Hemato-oncologic	0.551	0.818	0.321	0.773	0.601	0.695	1.000

*P* values are reported. Statistically-significant results are marked in bold

Renal stiffness was assessed for 19 patients by one trained operator through shear-waves elastography on Aixplorer (SuperSonic Imagine, Aix-en-Provence, France)

^a^Renal stiffness could not be evaluated in patients with pre-existing kidney comorbidities

## Discussion

Our study has described the epidemiology of chronic kidney disease and bone demineralization in HIV-positive patients from the Romanian HIV cohort. In this study 29.6 % of patients were diagnosed with stage 2 kidney disease and 1.4 % with stage 3 kidney disease. These numbers are quite high, compared with a prevalence of chronic kidney disease in patients with HIV infection reported at 6 % in the Danish HIV cohort study [9], 24 % in Nigeria [10], and 2 % [11], 10.2 % [12] or 15.5 % [13] in the United States of America.

We have also identified that cardiac comorbidities are risk factors for kidney disease in HIV-positive patients from our cohort (*p* = 0.013). Other similar studies also report hypertension as a risk factor for additional kidney damage in HIV-positive patients [11] compared with the HIV-negative population. Smoking has not been identified as a direct risk factor for renal disease (*p* = 0.109), our results being in line with data reported from other European countries, for example the Danish HIV cohort study [9]; however, given the recognized correlation between smoking and cardiovascular disease and our study’s findings that cardiac comorbidities associate a decreased eGFR, we suggest that HIV-positive patients who are current smokers should also be monitored for renal function. Moreover, as our study has shown that smoking is associated with significantly lower levels of hydroxy-vitamin D, this points to the fact that smoking should be avoided altogether by HIV-positive patients, as it not only targets the kidney, but the bone metabolism as well.

Lumbar osteopenia and osteoporosis were diagnosed in 33.3 and 13.7 % of patients, respectively; femoral osteopenia and osteoporosis were diagnosed in 37.3 and 7.8 %, respectively. Notably, the median age in this study was 38 years (IQR: 18 years), suggesting that this high prevalence of bone and kidney disease is rather correlated with the long evolution of HIV infection (median: 9 years, IQR: 13 years) than with patient age. These results are, to an extent, encouraging, as other studies report a higher prevalence of osteopenia or osteoporosis in HIV-positive patients, i.e., 51 and 10 %, respectively, in the United States of America [14], 63.2 and 15.1 % in Italy [15], and 56.5 and 10.7 % in Spain [16]. However, studies from Brazil report a much lower prevalence of decreased bone mineral density in patients with HIV infection, i.e. 14.6 % in the lumbar vertebrae and 5.6 % in the femur [17].

We also found lower nadir CD4 cell counts in patients with bone-related comorbidities (*p* = 0.000). A recent study from Australia defined a series of risk factors for low bone mineral density in treatment-naïve HIV-positive patients, and they found no association with nadir CD4 cell count [18]. However, seeing as that study was performed in patients with mean nadir CD4 cell counts above 500 cells/cmm, who had not yet met any criteria for starting ARV treatment, we expect their data to be significantly different from that derived from our study, where most patients were under ARV treatment, and the nadir CD4 cell count was 387 cells/cmm. Our finding that lower nadir CD4 cell counts are associated with bone-related abnormalities confirm previous studies performed in Spain, which reported lower bone mineral density in patients with lower nadir CD4 cell count [16].

## Conclusions

We identified a relatively high prevalence of chronic kidney disease in the Romanian HIV cohort, and a fairly low prevalence of osteopenia and osteoporosis, compared with other European countries. Lower nadir CD4 cell counts were statistically correlated with the presence of bone-related comorbidities. In this category of patients smoking should be avoided altogether, as it may be an indirect risk factor for kidney disease (associating cardiac comorbidities) and it may impair bone metabolism by altering serum levels of hydroxy-vitamin D.

## Abbreviations

ARV: antiretroviral
DXA: dual-energy X-ray absorptiometry
eGFR: estimated glomerular filtration rate
HAART: highly active antiretroviral therapy
IQR: interquartile range
SD: standard deviation
